# Reducing Medication Therapy Problems in the Transition from Hospital to Home: A Pre- & Post-Discharge Pharmacist Collaboration

**DOI:** 10.3390/pharmacy7030086

**Published:** 2019-07-09

**Authors:** Anne Schullo-Feulner, Lisa Krohn, Alison Knutson

**Affiliations:** 1 Park Nicollet Health Services, St. Louis Park, MN 55426, USA; 2 Pharmaceutical Care & Health Systems, University of Minnesota College of Pharmacy, Minneapolis, MN 55455, USA

**Keywords:** transitions in care, pharmacist, medication therapy problems, medication safety, comprehensive medication management, readmissions

## Abstract

Background: With 30-day Medicare readmission rates reaching 20%, a heightened focus has been placed on improving the transition process from hospital to home. For many institutions, this charge has identified medication-use safety as an area where pharmacists are well-positioned to improve outcomes by reducing medication therapy problems (MTPs). Methods: This system-wide (425 bed community hospital plus 18 primary care clinics) prospective study recruited inpatient and ambulatory pharmacists to provide comprehensive medication management before and after hospital discharge. The results analyzed were the success rate and timing of the inpatient to ambulatory pharmacist handoff, as well as the number, type, and severity of MTPs resolved in both settings. Results: Of the 105 eligible patients who received a pharmacist evaluation before discharge, 61 (58%) received follow-up with an ambulatory pharmacist an average of 2.88 days after discharge (range 1–8 days). An average of 5 and 1.4 MTPs per patient were identified and resolved in the inpatient vs. ambulatory setting, respectively. Although average MTP severity ratings were higher in the inpatient setting, the highest severity rating was seen most frequently in the ambulatory setting. Conclusions: In the transition from hospital to home, pharmacist evaluation in both the inpatient and ambulatory settings are necessary to resolve medication therapy problems.

## 1. Introduction

In the era of accountable care, organizations are charged with reaching quality metrics to optimize reimbursement. For some, this charge identified medication-use safety during transitions from hospital to home as an area for quality improvement [1,2,3]. Pharmacists in both inpatient and ambulatory settings have the potential to work together to improve medication-use safety by reducing medication therapy problems (MTPs) during the transition process [2,3,4]. One method to achieve this vision may be found in strategic alliances and collaboration across the care system to develop and evaluate a new model of pharmacy service.

With nearly one-fifth of hospitalized Medicare fee-for-service patients readmitted within 30 days, a heightened focus has been placed on improving the quality of the transition process to reduce costs and improve patient care [5]. Previous studies have demonstrated significant medication-related problems at the time of discharge, including mediation discrepancies that doubled readmission rates [6], as well as a lack of patient understanding, where 50% of patients were unable to state the name and purpose of their medications at the time of discharge [7]. One study found that over 50% of medication-related admissions were preventable, while another determined that these preventable admissions resulted in significantly longer hospital stays [8,9]. The Agency for Healthcare Research and Quality (AHRQ) recommends medication reconciliation, defined as the “comparison of the patient’s current medication regimen against the physician’s admission, transfer, and/or discharge orders to identify discrepancies,” as a beginning to improving medication safety [10]. Although this is necessary and provides the value of an accurate and complete medication list, it does not create a clinical assessment to ensure: (1) appropriate indication, (2) effectiveness, (3) safety, and (4) convenience/adherence for each medication. These four components provide the framework for the Patient-Centered Primary Care Collaborative model for comprehensive medication management (CMM) [11]. Through this assessment, pharmacists are able to identify, prevent, and/or resolve mediation therapy problems (MTPs). MTPs are defined as an event or circumstance involving drug treatment (pharmacotherapy) that interferes with the optimal provision of medical care [12]. Pharmacists are specifically trained to provide this comprehensive service, yet they remain underutilized during the transition process [13]. Previous scholarship substantiates pharmacist benefit, demonstrating that patients who met with a pharmacist either at the time of discharge or shortly after returning home have consistently lower readmission rates through the identification and resolution of MTPs [14,15,16,17,18,19,20]. The current study sought to build on this body of knowledge by evaluating a coordinated pharmacy service (CMM delivered by an inpatient pharmacist prior to discharge and an ambulatory pharmacist post-discharge) across the hospital to home transition of care. 

This paper describes the implementation and evaluation of a pharmacist-driven care transitions model in a large health care system. The specific objectives were to evaluate: (1) the success rate and timing of transferring patients from an inpatient to an ambulatory pharmacist, (2) the number and types of MTPs identified in the inpatient and ambulatory setting, and (3) the difference in MTP severity ratings (inpatient vs. ambulatory) for patients with a successful transfer of care.

## 2. Materials and Methods

This pharmacy-based care transition model was developed and conducted in a 425-bed community hospital and 18 primary care clinics within the Park Nicollet Health Services system in the Minneapolis-St Paul, Minnesota, United States of America (USA) metropolitan area. All pharmacists were employed by the health system, had full access to the electronic medical record, and were embedded in the care teams of their respective settings. Patients who received CMM from an inpatient pharmacist, ambulatory pharmacist, or both between August 2013 and November 2013 were included. Patients discharged to a skilled nursing facility or under hospice care were excluded from an ambulatory pharmacist evaluation but were still eligible to receive an inpatient pharmacist evaluation.

### 2.1. Care Transition Model Logistics

**Figure 1:**
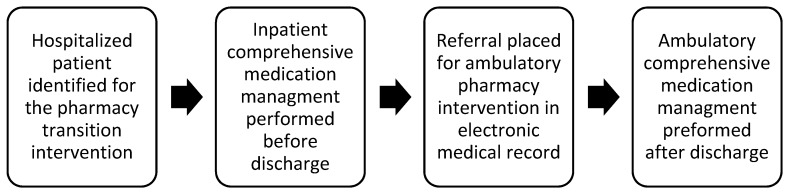
Care Transition Process.

Although discharging physicians were at liberty to request that any patient be included in the study, patients admitted with acute myocardial infarction, pneumonia, chronic obstructive pulmonary disease, and heart failure (i.e., U.S. Medicare Measures) were prioritized. Inpatient pharmacists completed a comprehensive medication management visit with the patient, as well as any caregivers present prior to discharge. The pharmacist discussed subsequent recommendations with the physician prior to the discharge medication ordering process. The pharmacy intervention was documented in the electronic medical record and sent with a referral for ambulatory pharmacy services to follow-up post discharge. As an integrated component of Park Nicollet’s medical home model, clinical ambulatory pharmacists are geographically dispersed around the region served. The post-discharge intervention consisted of an ambulatory CMM evaluation identical to that performed while the patient was hospitalized. This follow-up was performed either in person or by phone to reinforce inpatient recommendations, as well as identify and address any new MTPs that developed following discharge. See Figure 1 for a pictorial representation of the care transition process.

### 2.2. Clinical Services Provided 

Pharmacists in both settings collaborated with the care team (patient, nurse, physician, social work, etc.) to resolve MTPs and prepare a revised medication list. The inpatient care team helped to identify appropriate patients, as well as their anticipated date of discharge and discharge disposition. The team also provided additional information about the patient and current medical concerns. The revised medication list was created through calling pharmacies to review refill records, chart review, patient and/or family interview, and healthcare team collaboration. During the pharmacist visit, whether inpatient or ambulatory, a focus was placed on:

1. *Comprehensive Medication Management (CMM)*: Creating a medication list that is accurate across the transition, as well as ensuring each medication is indicated, effective, safe, and convenient with complete administration directions.
2. *Patient Centered Care*: Educating patients on a medication list developed in collaboration with patient needs and preferences.
3. *Medication Access*: Ensuring that patients have access to all medications and that each discharge prescription is sent to the correct pharmacy and/or location.

### 2.3. Data Collection/Research Methods

This study was given exemption status by Park Nicollet Health Services IRB and the University of Minnesota IRB. A total of 12 pharmacists, four inpatient and eight ambulatory, were trained to participate. Inpatient pharmacist documentation included both immediate resolution of MTPs and recommendations for consideration in the ambulatory setting. Ambulatory pharmacist documentation addressed inpatient recommendations, identified new MTPs since discharge, and provided a plan for continued ambulatory follow-up. In both settings, identified MTPs were categorized into eight types, consisting of: “medication inaccuracy,” “adverse drug reaction,” “non-adherence,” “lack of understanding,” “dose too high,” “lack of needed drug” (addition of a drug), “dose too low,” and “unnecessary medication” (unnecessary or duplicate medication). After an MTP was identified and typed, each one was given a severity rating from 1 (lowest) to 3 (highest/life-threatening) based on the potential for patient harm had the MTP not been resolved. Severity ratings for each intervention were documented based on reporting standards for skilled nursing facilities from the Department of Health and Human Services [21]; see Table 1 for detailed descriptions. Severity ratings were documented in real-time by the pharmacist but were also verified by three independent pharmacist raters via blind review after the study was completed. All raters were standardized using an inter-rater reliability test. When no MTP was identified, a severity rating of 0 was recorded.

### 2.4. Statistical Methods 

A generalized estimating equation (GEE) was chosen as a marginal model in order to account for the correlation of multiple measurements within some patients. An exchangeable correlation structure was specified, and a Gaussian distribution was chosen in order to compare the mean difference between the maximum severity ratings at ambulatory and inpatient evaluations. Additionally, paired t-tests comparing the inpatient and ambulatory severity ratings for only those patients with both interventions (i.e., successful transfer) were analyzed and the results were compared to those from the GEE analysis. Both models were implemented for the primary (overall severity) and secondary (individual MTPs rated for severity) analyses, with an α level of 0.05 considered significant. 

## 3. Results

All 12 trained pharmacists (four inpatient and eight ambulatory) participated. Patients were categorized based on having either a successful inpatient-to-ambulatory pharmacist transfer (n = 61) or having an inpatient evaluation only (n = 73). Baseline Characteristics (Table 2) between the two groups were well balanced with the following two exceptions. Patients who followed up with an ambulatory evaluation had shorter lengths of stay in the hospital (3.4 days compared to 5.1 days, *p*-value ≤ 0.001) and were more likely to discharge to home rather than a skilled nursing facility (*p*-value < 0.001). The average age, gender, marital status, number of chronic conditions, number of medications, and reason for evaluation were not found to be significantly different between the two groups. 

### 3.1. Success Rate of the Care Transition Model

Prior to discharge from the hospital, 134 patients received an inpatient pharmacist intervention. Of these 134 patients, 29 were discharged to a skilled nursing facility, excluding them from ambulatory follow-up. Of the patients eligible for an ambulatory intervention (n = 105), the transfer of care from the inpatient to the ambulatory setting was successful in 61 (58%) patients. In total, 44 eligible patients were lost to ambulatory follow-up due to: Inpatient pharmacist failure to place a transfer in the electronic system (44%); ambulatory pharmacist failure to act on a new patient transfer (31%); ambulatory pharmacist failure to reach the patient for follow-up (16%); patient readmission prior to ambulatory follow-up (7%); and lastly, patient declined ambulatory visit (2%). Excluding one outlier with a follow-up of 16 days, the average time to follow-up with an ambulatory pharmacist was 2.88 days (range 1–8 days).

### 3.2. Number and Types of Medication Therapy Problems Identified

An average of five MTPs per patient were identified and resolved in the inpatient setting prior to discharge. The most common types of MTP found in the inpatient setting were: “lack of needed drug” (identified in 60% of patients), “lack of understanding” (45% of patients), and “medication inaccuracy” (42% of patients). During the ambulatory follow-up, an average of 1.4 MTPs per patient were identified and resolved. In 75% of the patients who received both an inpatient and ambulatory intervention, the ambulatory pharmacist identified the presence of at least one MTP. During the ambulatory CMM intervention, medication “non-adherence” and “lack of needed drug” were the most common MTPs identified and resolved (each identified in 25% of patients), followed by “lack of understanding” (19.7% of patients) and “adverse drug reaction” (18.0% of patients). 

### 3.3. Severity Rating Comparison of Medication Therapy Problems Identified

Table 3 depicts the mean difference in maximum severity rating between the inpatient and ambulatory settings for patients with a successful transfer of care (i.e., the “inpatient and ambulatory CMM intervention cohort” n = 61). In terms of capacity to cause patient harm, all of the MTP types identified by pharmacists demonstrate a trend toward being less severe in the ambulatory vs. inpatient setting. When looked at as a whole, the mean difference in “overall MTP severity” decreased by 0.25 (CI: (−0.58, 0.08), *p*-value = 0.141) in the ambulatory setting, however, this difference did not reach significance. Four of the eight MTP types showed a similar nonstatistical trend toward reduced severity in the ambulatory setting. The other four MTP types (i.e., “medication inaccuracy,” “adverse drug reaction,” “dose too high,” and “lack of needed drug”), all demonstrated a statistically significant reduction in severity in the ambulatory vs. inpatient setting.

Table 4 depicts the frequency of each severity rating (0–3) for all patients in either the inpatient (n = 134) or ambulatory (n= 61) setting. There were a greater percentage of patients with no MTPs (identified as having a severity rating of “0”) in the ambulatory (26%) vs. inpatient setting (4%). Correspondingly, MTPs with severity ratings of 1 or 2 were seen more frequently in the inpatient setting. However, when it came to the highest severity rating (a rating of 3 indicating the potential for “permanent disability or death”), it was found more often in the ambulatory setting (found in 10% of patients in the ambulatory vs. 6% in the inpatient setting).

## 4. Discussion

Previous scholarship provides insight into the potential of pharmacists to improve medication safety via medication reconciliation in either the inpatient or ambulatory setting [2,3]. This study demonstrates a care model that created a successful direct handoff from inpatient pharmacists prior to discharge to ambulatory pharmacists’ post-discharge in 58% of eligible patients. The two most common reasons for failure (i.e., failure of inpatient pharmacist to place the transfer and failure of ambulatory pharmacist to follow-up on the transfer) primarily represented electronic and personnel system inefficiencies that have since been rectified. The inpatient process to initiate an ambulatory CMM intervention for a patient has been simplified down to three clicks in the electronic medical chart and can now be performed without a physician order by student pharmacists at the behest of a licensed pharmacist preceptor. On the ambulatory side, one FTE (full-time equivalent) of administrative personnel has been added to coordinate patient outreach in setting up either phone or in person visits for the health system wide ambulatory pharmacist group.

An average of five MTPs per patient were found in our inpatient cohort, while patients who received ambulatory follow-up only three days after discharge still had an average of 1.4 MTPs identified by this second CMM intervention. The benefit of comprehensive medication management both at the time of and directly after discharge has been demonstrated before [22,23]. One study found that two-thirds of patients discharged from an emergency department did not comprehend home care instructions, follow-up instructions, medications, and/or discharge diagnoses [23]. The current study supports this finding showing that despite receiving CMM just prior to discharge, nearly 75% of patients had at least one MTP at the ambulatory intervention. The presence of newly identified MTPs post-discharge demonstrates the need for comprehensive medication management at each transition in order to minimize medication related MTPs.

Although not statistically significant for all MTP types, this study demonstrated a reduction in overall MTP severity from the inpatient to ambulatory setting. This could be expected due to the acute illness of hospitalized patients. Perhaps more interesting is that while hospitalized patients had more severe MTPs, a greater percentage of severity level 3 (potential for severe harm) MTPs were found in the ambulatory setting. One possible explanation is that the level 3 MTPs identified in the ambulatory setting involved a lack of adherence to high-risk medications such as a hypoglycemic, opioid, or antithrombotic medication. Most commonly, non-adherence resulted from the patient preferring not to take, forgetting to take, an inability to afford the medications, and/or a lack of information retention post-discharge. Overall, data from this study shows that while more MTPs are found and resolved in the inpatient setting, those that persist and/or present themselves early in the ambulatory setting are of a more severe nature, emphasizing the importance of pharmacist intervention at both of these critical times during transitions in care.

There are several limitations to this study. Twelve pharmacists identified and classified MTPs. Although the pharmacy team underwent a norming process, there is still the potential for inter-rater variability. Similarly, MTP severity ratings were adjudicated by three separate normed judges. Further, the severity rating tool utilized was developed for use in long-term skilled nursing facilities, rather than hospitalized inpatients. This U.S. Department of Health and Human Services-based rating tool was selected by the health system’s Quality, Innovation, and Population Health Services department to align with other system-wide medication safety initiatives. Severity classification scales rating medication safety concerns in the inpatient [24,25] and outpatient [25] setting should be noted. There were limits on the discharge disposition type the ambulatory pharmacists were able to reach, as patients going to a skilled nursing facility were not seen across the transition. Due to the complexity of these patients’ medication regimens, follow-up with this population warrants further evaluation and is planned for the future. Also, due to the small sample size, this study was unable to show if pharmacy-based CMM reduced readmission rates. 

## 5. Conclusions

This study demonstrates a transition of care model for the transfer of patient care from an inpatient to an ambulatory pharmacist before and after discharge. This transition was successful in 58% of patients an average of 2.88 days after discharge. The two most common reasons for failure were failure of inpatient pharmacist to place the transfer and failure of ambulatory pharmacist to follow-up on the transfer. An average of five MTPs per patient were found in the inpatient cohort, and patients who received ambulatory follow-up roughly three days after discharge still had an average of 1.4 MTPs. Although overall MTP severity ratings were higher in the inpatient setting, MTPs that persisted and/or presented themselves early after discharge in the ambulatory setting were the most likely to lead to serious patient harm.

## Figures and Tables

**Table 1:** Potential Medication Therapy Problem Severity Rating.

Severity Rating	Description
1	If no intervention, potential for minimal (would require patient self-management) or no harm
2	If no intervention, potential for moderate harm (would require healthcare professional intervention or hospitalization to resolve)
3	If no intervention, potential for severe harm (permanent disability or death)

Note: Adapted from Medicare Nursing Home Levels of Harm categories.

**Table 2:** Baseline Characteristics.

Characteristic	Inpatient Intervention Only (n = 73)		Inpatient and Ambulatory Intervention (n = 61)		*p*-Value
Mean Age (years)	75.5		73.0		0.277
Sex					0.183
Female	36	(49.3%)	38	(62.3%)	
Male	37	(50.7%)	23	(37.7%)	
Marital Status					0.249
Single	17	(23.3%)	15	(24.6%)	
Married	26	(35.6%)	29	(47.5%)	
Divorced	2	(2.7%)	3	(4.9%)	
Widowed	28	(38.4%)	14	(23.0%)	
Mean Length of Stay (days)	5.1		3.4		<0.001
Mean Chronic Conditions (n)	9.2		10.6		0.076
Mean Chronic Medications (n)	12.5		13.5		0.290
Discharge Disposition					<0.001
Home	40	(54.8%)	56	(91.8%)	
Home (With Care)	4	(5.5%)	5	(8.2%)	
Skilled Nursing Facility	29	(39.7%)	0	(0%)	
Insurance					0.456
Private	5	(6.8%)	6	(9.8%)	
Medicare	62	(84.9%)	50	(82.0%)	
Medicaid	5	(6.8%)	2	(3.3%)	
None	1	(1.4%)	3	(4.9%)	
Reason for Evaluation *					0.498
COPD	17		11		
MI	4		2		
Pneumonia	8		11		
CHF	13		16		
Other	36		26		

* Patients could contribute to multiple categories.

**Table 3:** Medication Therapy Problem Severity Comparison for the Inpatient and Ambulatory CMM Intervention Cohort (n = 61) *.

MTP Severity Rating	Mean Difference in Inpatient vs. Ambulatory Maximum Severity	95% CI	*p*-Value
Overall Severity	−0.25	(−0.58, 0.08)	0.141
Medication Inaccuracy	−0.34	(−0.58, −0.11)	0.004
Adverse Drug Reaction	−0.33	(−0.63, −0.02)	0.036
Non-Adherence	−0.20	(−0.47, 0.08)	0.159
Lack of Understanding	−0.23	(−0.53, 0.07)	0.137
Dose Too High	−0.34	(−0.55, −0.14)	0.001
Lack of Needed Drug	−0.46	(−0.75, −0.17)	0.002
Dose Too Low	−0.07	(−0.32, 0.19)	0.610
Unnecessary Medication	−0.18	(−0.38, 0.02)	0.070

* Represents only patients receiving both an inpatient and ambulatory pharmacist evaluation (n = 61).

**Table 4:** Frequency of Severity Ratings.

Severity Rating	Inpatient (n = 134)		Ambulatory (n = 61)	
0	5	(3.7%)	16	(26.2%)
1	29	(21.6%)	7	(11.5%)
2	92	(68.7%)	32	(52.5%)
3	8	(6.0%)	6	(9.8%)

## References

[1] Johansen J.S., Havnes K., Halvorsen K.H., Haustreis S., Skaue L.W., Kamycheva E., Mathiesen L., Viktil K.K., Granås A.G., Garcia B.H. (2018). Interdisciplinary collaboration across secondary and primary care to improve medication safety in the elderly (IMMENSE study): Study protocol for a randomized controlled trial. BMJ Open.

[2] Hohl C.M., Partovi N., Ghement I., Wickham M.E., McGrail K., Reddekopp L.N., Sobolev B. (2017). Impact of early in-hospital medication review by clinical pharmacists on health services utilization. PLoS ONE.

[3] Martin P., Tamblyn R., Benedetti A., Ahmed S., Tannenbaum C. (2018). Effect of a Pharmacist-Led Educational Intervention on Inappropriate Medication Prescriptions in Older Adults: The D-PRESCRIBE Randomized Clinical Trial. JAMA.

[4] Dudas V., Bookwalter T., Kerr K., Pantilat S. (2001). The impact of follow-up telephone calls to patients after hospitalization. Am. J. Med..

[5] Unruh M.A., Jung H.Y., Vest J.R., Casalino L.P., Kaushal R. (2017). Meaningful Use of Electronic Health Records by Outpatient Physicians and Readmissions of Medicare Fee-for-Service Beneficiaries. Med. Care.

[6] Coleman E.A., Smith J.D., Raha D., Min S.J. (2005). Posthospital medication discrepancies: Prevalence and contributing factors. Arch. Intern. Med..

[7] Makaryus A.N., Friedman E.A. (2005). Patients understanding of their treatment plans and diagnosis at discharge. Mayo Clin. Proc..

[8] Winterstein A.G., Sauer B.C., Hepler C.D., Poole C. (2002). Preventable drug-related hospital admissions. Ann. Pharm..

[9] Dormann H., Neubert A., Criegee-Rieck M., Egger T., Radespiel-Tröger M., Azaz-Livshits T., Hahn E.G. (2004). Readmissions and adverse drug reactions in internal medicine: The economic impact. J. Intern. Med..

[10] Agency for Healthcare Research and Quality (AHRQ): Medications at Transitions and Clinical Handoffs (MATCH) Toolkit for Medication Reconciliation. http://www.ahrq.gov/qual/match/.

[11] Patient-Centered Primary Care Collaborative Resource Guide: The Patient-Centered Medical Home: Integrating Comprehensive Medication Management to Optimize Patient outcomes. http://www.pcpcc.org/sites/default/files/media/medmanagement.pdf.

[12] Strand L.M., Morley P.C., Cipolle R.J., Ramsey R., Lamsam G.D. (1990). Drug-related problems: Their structure and function. DICP Ann. Pharmacother..

[13] Kern K.A., Kalus J.S., Bush C., Chen D., Szandzik E.G., Haque N.Z. (2014). Variations in pharmacy-based transition-of-care activities in the United States: A national survey. Am. J. Health Syst. Pharm..

[14] Bellone J.M., Barner J.C., Lopez D.A. (2012). Postdischarge interventions by pharmacists and impact on hospital readmission rates. J. Am. Pharm. Assoc..

[15] Conklin J.R., Togami J.C., Burnett A., Dodd M.A., Ray G.M. (2014). Care transitions service: A pharmacy-driven program for medication reconciliation through the continuum of care. Am. J. Health Syst. Pharm..

[16] Monika G., Mikaitis D., Gayle S., Johnson T., Sims S. (2013). Impact of a combined pharmacist and social worker program to reduce hospital readmissions. J. Manag. Care Pharm..

[17] Kilcup M., Schultz D., Carlson J., Wilson B. (2013). Postdischarge pharmacist medication reconciliation: Impact on readmission rates and financial savings. J. Am. Pharm. Assoc..

[18] Kripalani S., Roumie C.L., Dalal A.K., Cawthon C., Businger A., Eden S.K., Huang R.L. (2012). Effect of a pharmacist intervention on clinically important medication errors after hospital discharge: A randomized trial. Ann. Intern. Med..

[19] Pal A., Babbott S., Wilkinson S.T. (2013). Can the targeted use of a discharge pharmacist significantly decrease 30-day readmissions?. Hosp. Pharm..

[20] Mekonnen A.B., McLachlan A.J., Brien J.A. (2016). Effectiveness of pharmacist-led medication reconciliation programmes on clinical outcomes at hospital transitions: A systematic review and meta-analysis. BMJ Open.

[21] Medicare.gov Medicare Nursing Home Levels of Harm Categories. Http://www.medicare.gov/NHCompare/static/related/incdrawlevelofharm.asp?language=English&version=default.

[22] Phatak A., Prusi R., Ward B., Hansen L.O., Williams M.V., Vetter E., Chapman N., Postelnick M. (2016). Impact of pharmacist involvement in the transitional care of high-risk patients through medication reconciliation, medication education, and postdischarge call-backs (IPITCH Study). J. Hosp. Med..

[23] Engel K.G., Buckley B.A., Forth V.E., McCarthy D.M., Ellison E.P., Schmidt M.J., Adams J.G. (2012). Patient understanding of emergency department discharge instructions: Where are knowledge deficits greatest?. Acad. Emerg. Med..

[24] Hartwig S.C., Siegel J., Schneider P.J. (1992). Preventability and severity assessment in reporting adverse drug reactions. Am. J. Hosp. Pharm..

[25] National Coordinating Council for Medication Error Reporting and Prevention (NCC MERP) Taxonomy of Medication Errors. http://www.NCCMERP.org.

